# Algal Blooms in Lakes Increase After Wildfire Smoke Events in the Contiguous United States

**DOI:** 10.1002/gcb4.70004

**Published:** 2026-03

**Authors:** Nicole E. Olson, Meredith M. Brehob, Robert D. Sabo, Irena Pavlovic, Kathleen I. Shank, Sam Penry, Amalia M. Handler, Michael J. Pennino, R. Byron Rice, Katie L. Boaggio, Stephen D. LeDuc

**Affiliations:** 1 U.S. Environmental Protection Agency, Office of Research and Development, Research Triangle Park, North Carolina, USA; 2 Oak Ridge Institute for Science and Education Hosted at U.S. Environmental Protection Agency, Office of Research and Development, Washington D.C., USA; 3 U.S. Environmental Protection Agency, Office of Research and Development, Washington D.C., USA; 4 U.S. Environmental Protection Agency, Region 9 Air and Radiation Division, Southern California Field Office, California, USA; 5 Oak Ridge Associated Universities Hosted at U.S. Environmental Protection Agency, Office of Research and Development, Research Triangle Park, North Carolina, USA; 6 U.S. Environmental Protection Agency, Office of Research and Development, Corvallis, Oregon, USA; 7 U.S. Environmental Protection Agency, Office of Air and Radiation, Research Triangle Park, North Carolina, USA

**Keywords:** algal bloom, chlorophyll-a, cyanobacteria, drinking water, lakes, nutrients, remote sensing, smoke, wildfire

## Abstract

Area burned by wildfire has increased in the contiguous United States and in many places globally in recent years, impacting communities and ecosystems nearby and even far downwind of fires. We examined the potential effects of smoke on surface chlorophyll-a concentrations in lakes, as algal blooms can adversely impact drinking water supplies and aquatic ecosystems. We linked remotely sensed surface chlorophyll-a concentrations from over 2000 lakes across the contiguous U.S. with airborne smoke plume locations and densities for 2018. Chlorophyll-a values for each lake were fitted to seasonal curves, and the residuals were tested between smoke versus no smoke periods. Changes in chlorophyll-a were also compared between the weeks before and after smoke events for each lake. Lastly, we categorized lakes based on elevation and trophic status to further discern fire effects on water quality. Mean residual surface chlorophyll-a concentrations during and within 2 weeks after smoke periods were significantly elevated (1.5 μg/L) compared to values from no smoke periods (−0.3 μg/L). Moreover, surface chlorophyll-a responses increased significantly with higher smoke density, and in hundreds of the lakes, increased by amounts exceeding global drinking water alerts for chlorophyll-a. We observed significant increases in responses for lakes at higher elevation (> 538 m) and for eutrophic lakes. We hypothesize that fire-driven smoke effects are contributing to the formation of algal blooms given the proper antecedent conditions, like elevated water temperatures. Algal blooms can occur after ephemeral change in lake trophic status and produce cyanotoxins and other adverse impacts. Because wildfire smoke can affect large regions, our findings have implications for drinking water and recreational lakes across the U.S. and globally, as well as for lake ecology, particularly in higher elevation systems with otherwise limited nutrient inputs.

## 1 | Introduction

In recent decades, the western United States and other regions globally have experienced an increase in area burned by wild-fire (Abatzoglou and Williams 2016; Burton et al. 2024; Juang et al. 2022; Senande-Rivera et al. 2022). Besides risks to life and property, this increase in wildfire activity has resulted in degradation of air and water quality. Increases in smoke from wildfires have reversed trends in improvements in airborne fine particulate matter (PM_2.5_) concentrations in the western U.S. (Burke et al. 2023), with profound implications for human health (U.S. EPA 2019, 2022). Fires also mobilize heavy metals and other hazardous air pollutants, both locally and downwind of the fire epicenter (Boaggio et al. 2022; Rice et al. 2023). Concomitantly, numerous studies have shown the effects of wildfire and subsequent erosion on local water quality in burned watersheds (Paul et al. 2022; Pennino et al. 2022; Rhoades et al. 2019; Rust et al. 2018). Metals and nutrients, among other constituents, are often elevated in downstream waterbodies for several years or more post-fire (Paul et al. 2023), and the use of fire retardants can contribute to nutrient loading to watersheds (Moorhead et al. 2025). Despite these well-recognized impacts, there are aspects of increased wildfire burning that remain relatively poorly understood and require further study.

In stark contrast to local water quality effects, the potential for downwind impacts of wildfire smoke on ecosystems, especially waterbodies, is only now being recognized (Farruggia et al. 2024; Paul et al. 2023). Recently, studies have shown that wildfire smoke can attenuate light, thus temporarily affecting respiration, water temperature, and the movement of fish, among other aquatic impacts (David et al. 2018; Scordo et al. 2021; Smits et al. 2024). It has also been increasingly recognized that particles and ash in wildfire smoke are often nutrient-rich, creating the potential for downwind deposition onto waterbody surfaces (Fernandez et al. 2024; Koplitz et al. 2021; Olson et al. 2023).

Indeed, in a previous study, we observed significant increases in atmospheric concentrations of nutrients on wildfire smoke days in the western U.S., with some maximum daily concentrations exceeding daily non-smoke averages by several orders of magnitude (Olson et al. 2023). Phosphorus was especially elevated on smoke-impacted days. For example, one air monitor recorded a phosphorus measurement on a smoke-impacted day that was more than 85,000% higher than the yearly non-smoke average value at that monitor. In a few cases, the presence of smoke has been linked to increases in phosphorus in the water column, with generally positive correlations between atmospheric phosphorus deposition and total phosphorus concentrations in rivers and streams (Fernandez et al. 2024; Sabo et al. 2023; Spencer and Hauer 1991), although the ecosystem effects of this increase remain unstudied.

One potential impact of wildfire additions of phosphorus and other nutrients to downwind waterbodies may be the proliferation of algal blooms. Harmful algal blooms (HABs) can negatively affect aquatic life, recreation, and drinking water quality (Brooks et al. 2016). Among other effects, blooms can create zones of low dissolved oxygen, potentially resulting in mortality of fish and other aquatic organisms (Smith et al. 1999). Often referred to as “cyanoHABS”, certain HAB formations contain high concentrations of cyanobacteria, a type of photosynthetic bacteria that can produce cyanotoxins harmful to humans, pets, or livestock (Paerl et al. 2001). HABs and cyanoHABS are prevalent in watersheds with high external nutrient inputs, such as agriculturally dominated watersheds (Brehob et al. 2024; Iiames et al. 2021). But HABs have also been recorded in relatively pristine, remote lakes in the absence of high nutrient inputs (Reinl et al. 2021). Nutrient deposition via wildfire smoke could contribute to the onset of HAB events, especially in higher elevation lakes with otherwise low nutrient inputs (Jansen et al. 2024). Indeed, we observed a correlation in time between the presence of wildfire smoke and cyanobacteria bloom intensity in a small number of lakes in the same study where we found phosphorus and other nutrients to be significantly elevated on smoke-impacted days (Olson et al. 2023). However, the number of lakes analyzed via remote sensing (*n* = 10) was too small to draw firm conclusions about the relationship between wildfire smoke and cyanobacteria activity.

Here, we examine the potential for this smoke-algal bloom relationship using remote sensing data for both wildfire smoke plumes and cyanobacteria activity in over 2000 lakes in the contiguous U.S. (CONUS). Specifically, using remote sensing derived estimates of surface chlorophyll-a concentrations as a measure of algal bloom activity and focusing on 2018 data, we address the following questions:

1. Is the presence of wildfire smoke linked to a significant increase in surface chlorophyll-a, representative of algal biomass and cyanobacteria blooms?
2. Does denser smoke lead to a significantly greater surface chlorophyll-a response compared to lower levels of smoke?
3. Are surface chlorophyll-a responses to smoke greater in higher elevation lakes relative to lower elevation lakes?
4. Are surface chlorophyll-a responses to smoke greater in nutrient-poor (i.e., oligotrophic) lakes or already nutrient-rich (i.e., eutrophic) lakes?

Answers to these questions will provide resource managers greater ability to anticipate the occurrence of algal blooms, especially given that wildfire activity is predicted to increase in the near future.

## 2 | Materials and Methods

### 2.1 | Smoke Plume Identification

**FIGURE 1 |:**
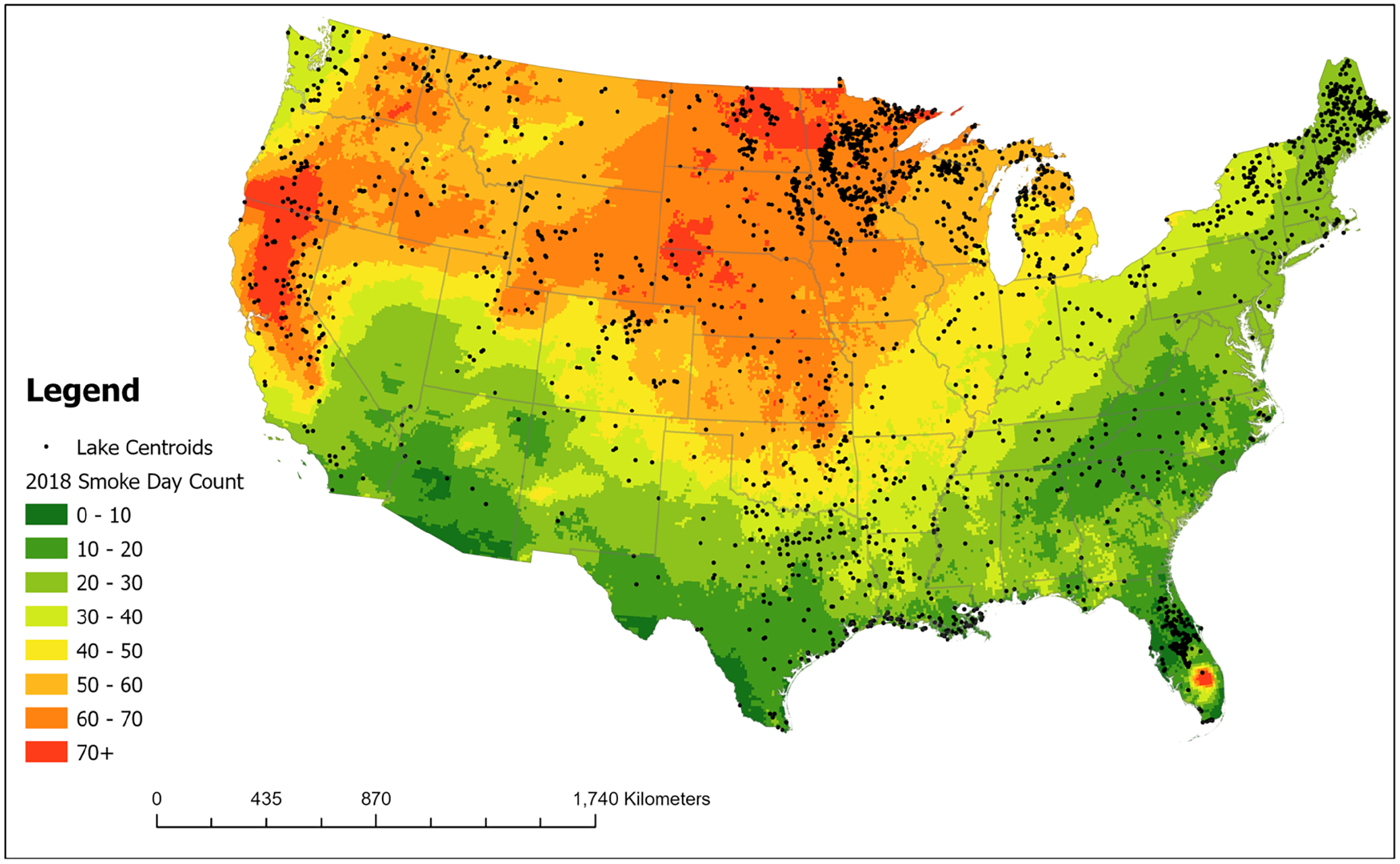
Map of the CONUS showing total smoke days in 2018. Points represent lake centroids that indicate lakes resolvable by the Cyanobacteria Assessment Network (CyAN) satellites.

To characterize smoke days, the publicly available Hazard Mapping System (HMS) smoke product (https://www.ospo.noaa.gov/Products/land/hms.html) from the National Oceanic and Atmospheric Administration (NOAA) was used to identify daily smoke plumes in the atmospheric column (Ruminski et al. 2006, 2007). HMS has been extensively validated by prior studies (Brey et al. 2018; Larsen et al. 2018). The smoke density was assigned arbitrary values of 5, 16, or 27 μg/m^3^; which were defined as low-, medium-, and high-density, respectively. Where smoke polygons overlapped in the dataset, the highest density value was chosen for that day following methods used previously (Olson et al. 2023; Smits et al. 2024). Cumulative smoke days for the year 2018 are shown in a map of the CONUS in Figure 1.

### 2.2 | Satellite Surface Chlorophyll-a Measurements

For this study, the HMS smoke product was aggregated with remote sensing cyanobacteria data to compare surface chlorophyll-a concentrations before, during, and after contact with wildfire smoke. To characterize algal blooms in lakes, we used weekly data from the Ocean and Land Colour Instrument (OLCI) onboard the Sentinel-3A/B satellites from 1/1/2018–12/30/2018. This resulted in 68,992 rows of data. These satellites have a combined revisit frequency of 1–2 days and images have a pixel resolution of 300 m × 300 m (Coffer, Schaeffer, Salls, et al. 2021). Pixels flagged due to issues such as glint, mixed land and water, cloud cover, or cloud shadow are removed. Pixels that cover the land/water boundary and are land-adjacent are removed (Urquhart and Schaeffer 2020). There are a total of 2196 waterbodies of sufficient size and shape that contain at least three reliably resolvable pixels within the lake boundary across the CONUS (Figure 1) (Urquhart and Schaeffer 2020). These lakes range in size from approximately 0.75 km^2^ to over 4000 km^2^ (Coffer et al. 2020).

The U.S. Environmental Protection Agency’s (EPA’s) Cyanobacteria Assessment Network (CyAN, https://www.epa.gov/water-research/cyanobacteria-assessment-network-cyan; Schaeffer et al. 2015) processes the satellite imagery to calculate the cyanobacteria index (Coffer et al. 2020), a relative measure of cyanobacteria in lake water (Schaeffer et al. 2015; Seegers et al. 2021). The cyanobacteria index is calculated based on the spectral shape at and adjacent to 681 nm which covers the spectral range associated with chlorophyll-a and phycocyanin absorption and fluorescence (Kutser 2009; Sharp et al. 2021; Wynne et al. 2008). Chlorophyll-a is a photosynthetic pigment found in all phytoplankton, whereas phycocyanin is a photosynthetic pigment specific to cyanobacteria (commonly referred to as blue-green algae) (Coffer et al. 2020; Seegers et al. 2021; Wynne et al. 2008). The cyanobacteria index was optimized to detect the spectral signature of cyanoHABs (which includes leveraging chlorophyll spectral signatures of all algal species and other wavelengths specific to phycocyanin), but novel detection methods and algorithms have been generally optimized to estimate chlorophyll-a concentrations across a variety of lakes with different tropic statuses (Coffer, Schaeffer, Salls, et al. 2021; Seegers et al. 2021). Studies comparing satellite cyanobacteria index measurements with in situ conditions show good agreement (Mishra et al. 2021; Schaeffer et al. 2018; Seegers et al. 2021; Whitman et al. 2022).

We used near-daily satellite measurements aggregated into 7 day composites, which preserved the maximum data value for each pixel collected in the 7 day window. For each lake, we converted the cyanobacteria index to surface chlorophyll-a concentration according to (Seegers et al. 2021) and (Coffer et al. 2020). We calculated mean surface chlorophyll-a concentration in the lake by summing all resolved pixel data values and dividing by the number of resolved pixels, resulting in area-normalized chlorophyll-a from cyanobacteria (Mishra et al. 2019). The use of surface chlorophyll-a concentrations now allows us to directly relate these concentrations to shifts in nutrient criteria, and more fully communicate general risk of HABs and cyanoHABs. CyAN and HMS smoke plume data were joined by date and latitude/longitude in R (R Development Core Team 2024).

### 2.3 | Smoke Pattern Identification for Spline Analysis

Each satellite-resolved lake was evaluated for patterns of intersection with atmospheric smoke polygons. Smoke data were aggregated to the same weekly intervals as the CyAN data, such that the highest smoke density value for the 7 day period was preserved (Smits et al. 2024). A smoke event was defined as any week in which a smoke polygon was detected over the lake boundary. A no smoke event is defined as any week for which a smoke polygon was not detected over the lake boundary. Smoke-impacted lakes were defined as lakes that contained at least one instance of a no smoke event, followed by a smoke event, followed by a no smoke event, in that order. Lakes were considered non-smoke impacted if they had six or more weeks of no smoke detected overhead. Missing smoke event data values were kept and labeled as NA. Analyses were conducted in R (R Development Core Team 2024).

Data were joined with contextual environmental data for each lake. Elevation data were gathered from the U.S. EPA’s LakeCat dataset (https://www.epa.gov/national-aquatic-resource-surveys/lakecat-dataset) (Hill et al. 2018), a publicly available dataset of lake metrics. Any missing values were defined as NA and not removed from the dataset. Data subsets (oligotrophic (*n* = 435, chlorophyll-a < 2 μg/L), mesotrophic (*n* = 121, chlorophyll-a 2–7 μg/L), eutrophic (*n* = 348, chlorophyll-a > 7 μg/L), lower elevation (*n* = 111, < 255 m), medium elevation (*n* = 576, 255–538 m) and higher elevation (*n* = 217, > 538 m)) were determined based on EPA trophic status guidelines (U.S. EPA 2009) and by calculating the lowest, middle two, and highest quartiles of elevation from the population of lakes with smoke events. To account for the seasonal correlation between bloom magnitude and smoke occurrence, data were broken into three seasons (spring, summer, and fall; Figure S1). For each lake and season, the difference between the average bloom magnitude during smoke weeks and average bloom magnitude during non-smoke weeks was calculated. Then the 95% confidence interval across lakes was determined. In all seasons, the 95% confidence interval was positive and did not overlap with zero, indicating a tendency for bloom magnitude to be higher during smoke weeks. These analyses were executed in R v4.4.0 (R Development Core Team 2024).

### 2.4 | Spline/Residual Analysis

**FIGURE 2 |:**
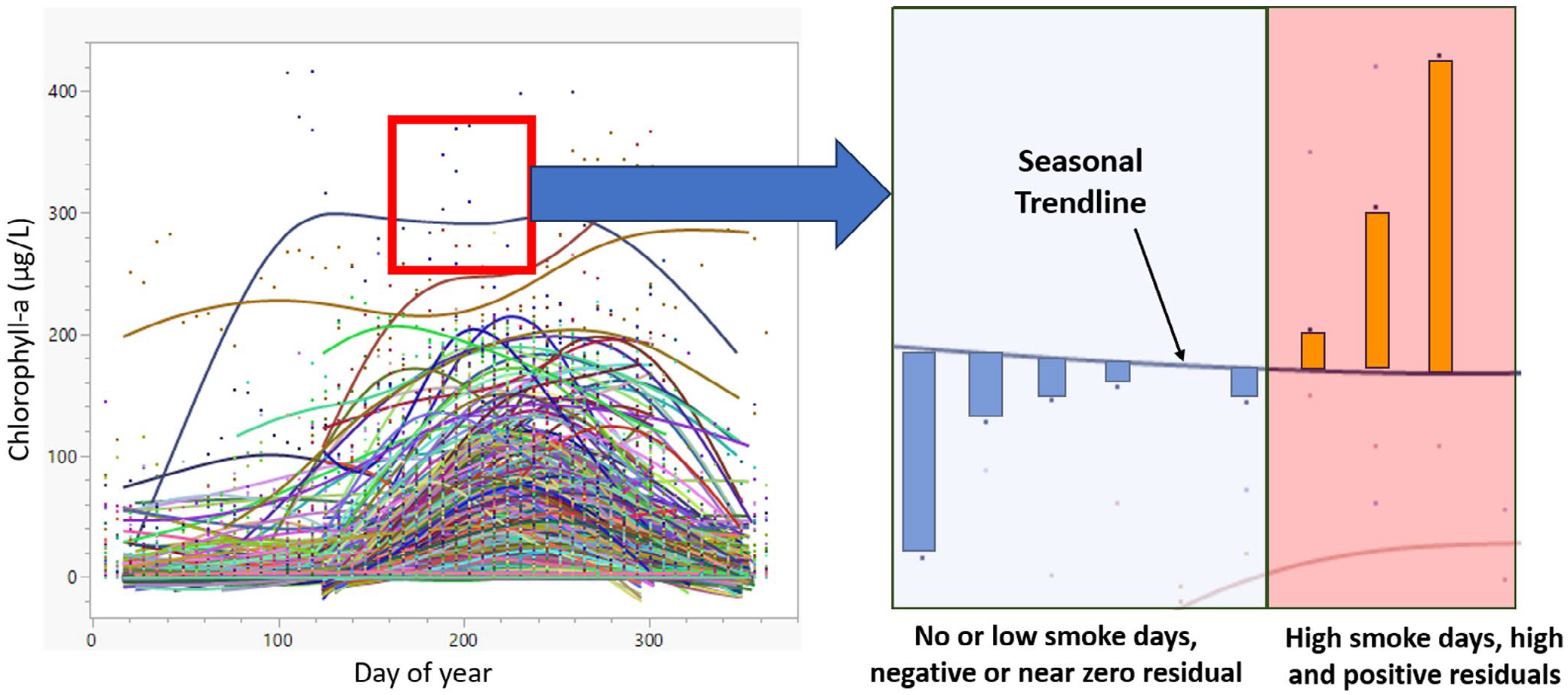
Depiction of residuals used in the spline regression to account for seasonal bloom dynamics. Each fitted line represents an individual lake, while data points indicate observed surface chlorophyll-a values. Orange bars indicate positive residuals (i.e., surface chlorophyll-a values that were higher than predicted based on seasonal trends); blue bars indicate negative residuals (i.e., lower than predicted surface chlorophyll-a values). Weekly residuals were statistically tested with ANOVA at significance levels of *p* < 0.05. Refer to the methods section 2.4 for additional details.

Individual time series of observed surface chlorophyll-a concentrations were fitted with splines to approximate the seasonal dynamics of bloom phenology for an individual lake (Figure S1; *λ* = 1.0, trim = 0.6). Following spline fitting, residuals were calculated by subtracting spline predicted surface chlorophyll-a values from observed values (Figure 2). Positive residuals indicated a tendency for surface chlorophyll-a to be higher than predicted, often occurring during smoke events. Negative residuals described a smaller-than-predicted surface chlorophyll-a concentration, usually but not always occurring during periods of low or no smoke. Weekly residuals were then subjected to two separate ANOVA tests to evaluate the effects of smoke on the mean residual deviation from the overall seasonal pattern for surface chlorophyll-a. The first ANOVA test evaluated the impacts of smoke events of any smoke density compared to no smoke events. The second test assessed the impact of smoke density (no smoke/no observation taken, or low-, medium-, and high-density smoke) on the mean residual variance. If a significant difference in treatments was detected (*p* < 0.05), post hoc pairwise comparisons were executed using Student’s *t* test. Residuals were also summarized and visualized via histograms, and specific percentile comparisons were highlighted across treatments (Figure S2). Relative residuals were calculated by taking the observed chlorophyll-a divided by the spline predicted chlorophyll-a. All analyses were executed in JMP-PRO 17.

## 3 | Results

**FIGURE 3 |:**
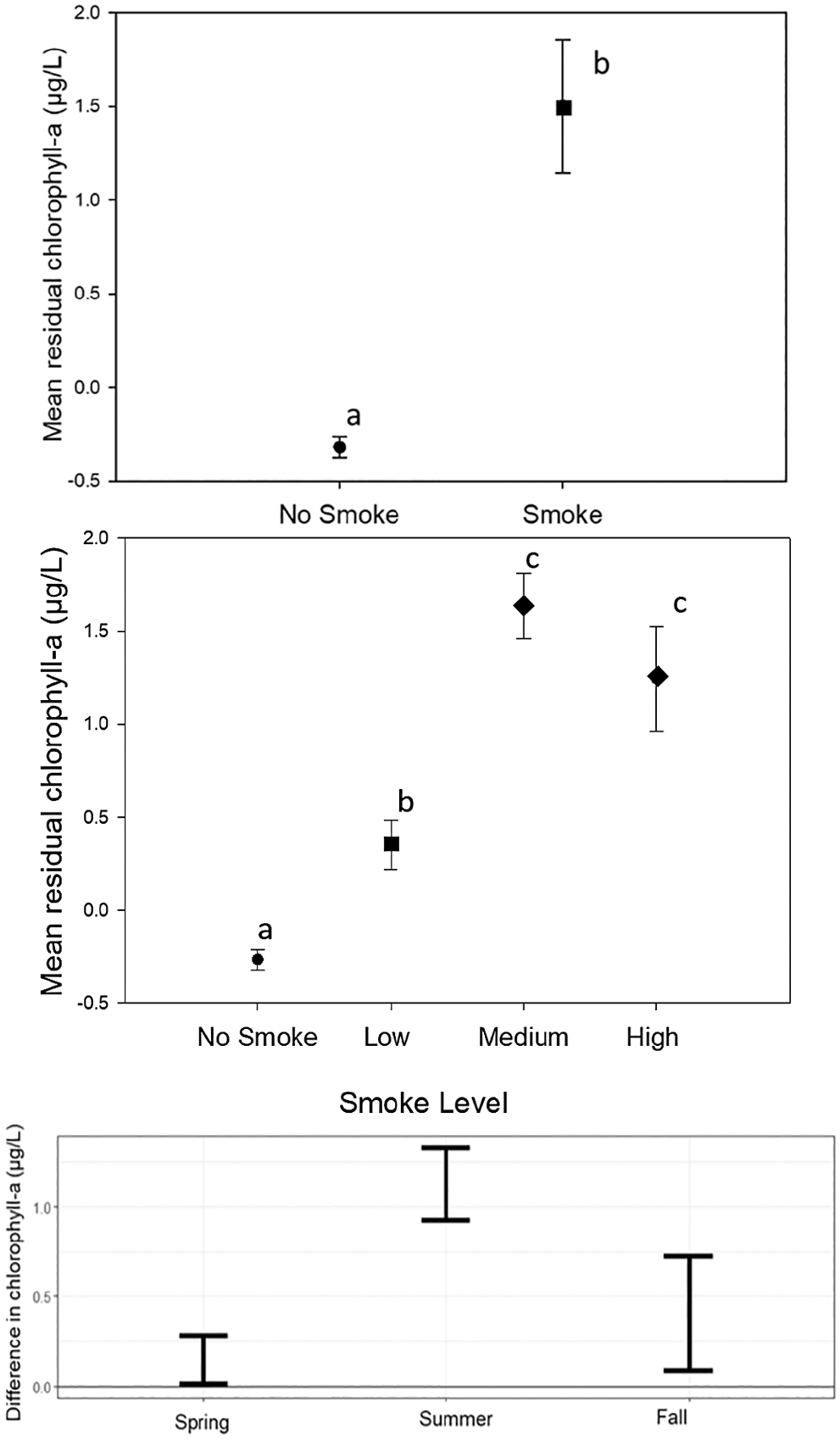
Average residual surface chlorophyll-a values during smoke and no smoke events (top) and during different density smoke categories (middle). Residuals are from spline regressions of surface chlorophyll-a versus time of year for each individual lake. Mean values are plotted with bars representing standard error. Letters denote data significantly different (*p* < 0.05). Data from these plots, including sample sizes, are shown in Tables S2 and S3. The 95% confidence intervals of the seasonal difference between surface chlorophyll-a during smoke and non-smoke weeks across all lakes (bottom). In all seasons, the 95% confidence interval was positive and did not overlap with zero, indicating a tendency for surface chlorophyll-a to be higher during smoke weeks.

Surface chlorophyll-a residuals were significantly higher during smoke event weeks (1.5 ± 0.50 μg/L) compared to weeks with no smoke (−0.3 ± 0.05 μg/L, Figure 3, ANOVA *p* < 0.05). We further classified smoke density by no smoke or low-, medium-, or high-density smoke. We found significant differences in residual surface chlorophyll-a concentrations for lakes experiencing medium- (1.6 ± 0.17 μg/L) or high-density smoke weeks (1.2 ± 0.28 μg/L) events when compared to low-density (0.3 ± 0.13 μg/L) or no smoke weeks (−0.3 ± 0.05 μg/L). Thus, we observed a significant association between the presence of wildfire smoke and increased surface chlorophyll-a in individual lakes, even when accounting for seasonal differences in chlorophyll-a (Figure 3 (bottom); Figure S1).

**FIGURE 4 |:**
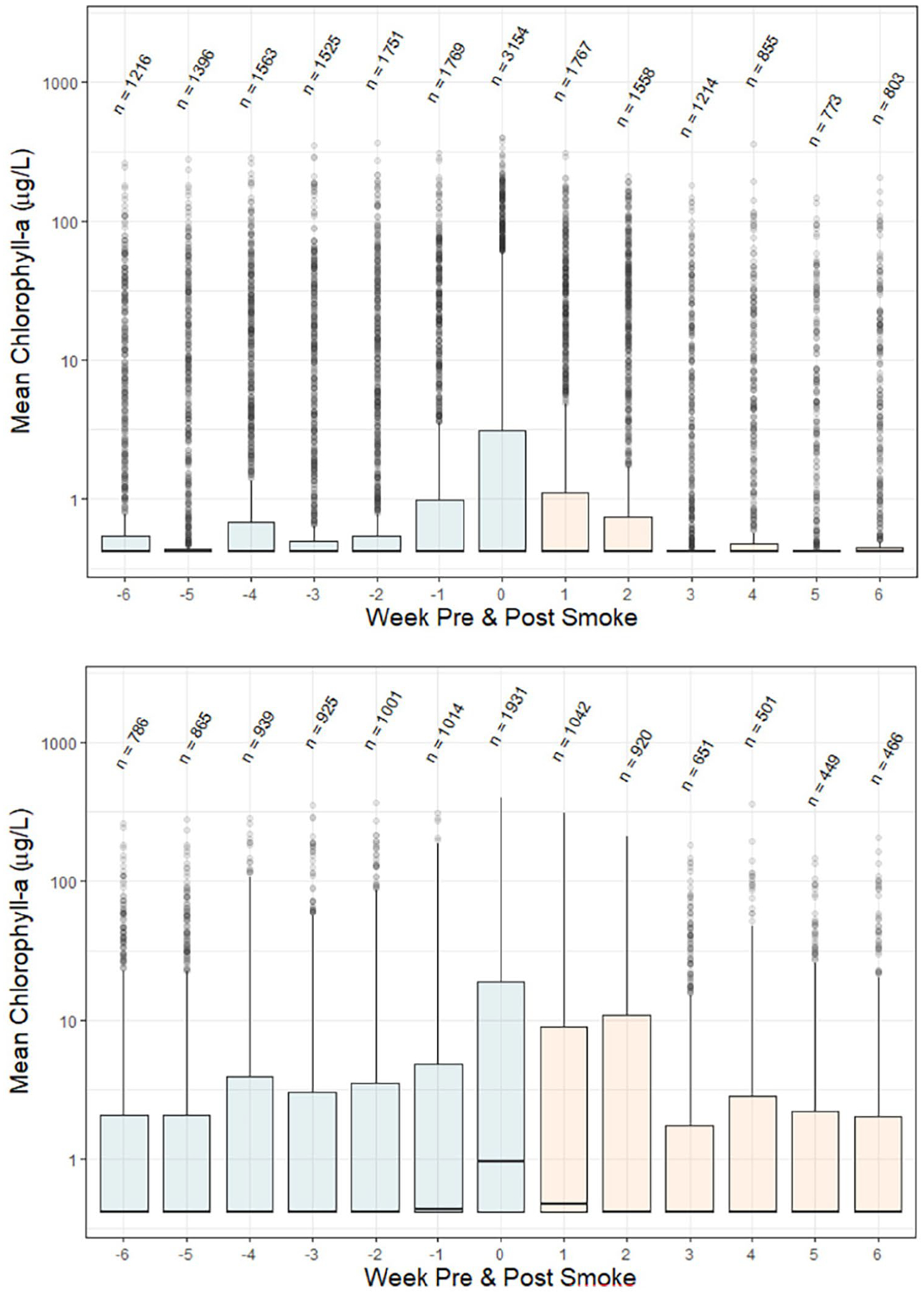
Average weekly chlorophyll-a for smoke impacted lakes. Week 0 indicates a smoke event, with negative weeks representing the weeks prior to smoke exposure (colored blue) and positive weeks indicating weeks after smoke exposure (colored orange). The plot on the top contains all data; the plot on the bottom removed lakes with > 80% of observations at the method detection limit to enable easier visualization of trends. Medians are shown in black lines; numbers of measurements are listed above each bar. Corresponding data are shown in Table S1.

Comparison of lakes with smoke events showed that lakes affected by smoke experienced higher maximum surface chlorophyll-a concentrations (400 μg/L during the smoke event compared to 310 μg/L the week before smoke exposure; Figure 4 and Table S1). Comparison of medians across the dataset did not yield different results, likely because so many measurements occurred at the limit of detection. To further explore this trend, we removed lakes where > 80% of measurements were at the method limit of detection. With this approach, we were able to see the median chlorophyll-a concentration was higher (0.95 μg/L) during a week with smoke compared to the weeks prior (0.4 μg/L; Figure 4 and Table S1). Surface chlorophyll-a remained elevated for 1–2 weeks following the smoke event before returning to levels similar to or even lower than before the smoke event.

**FIGURE 5 |:**
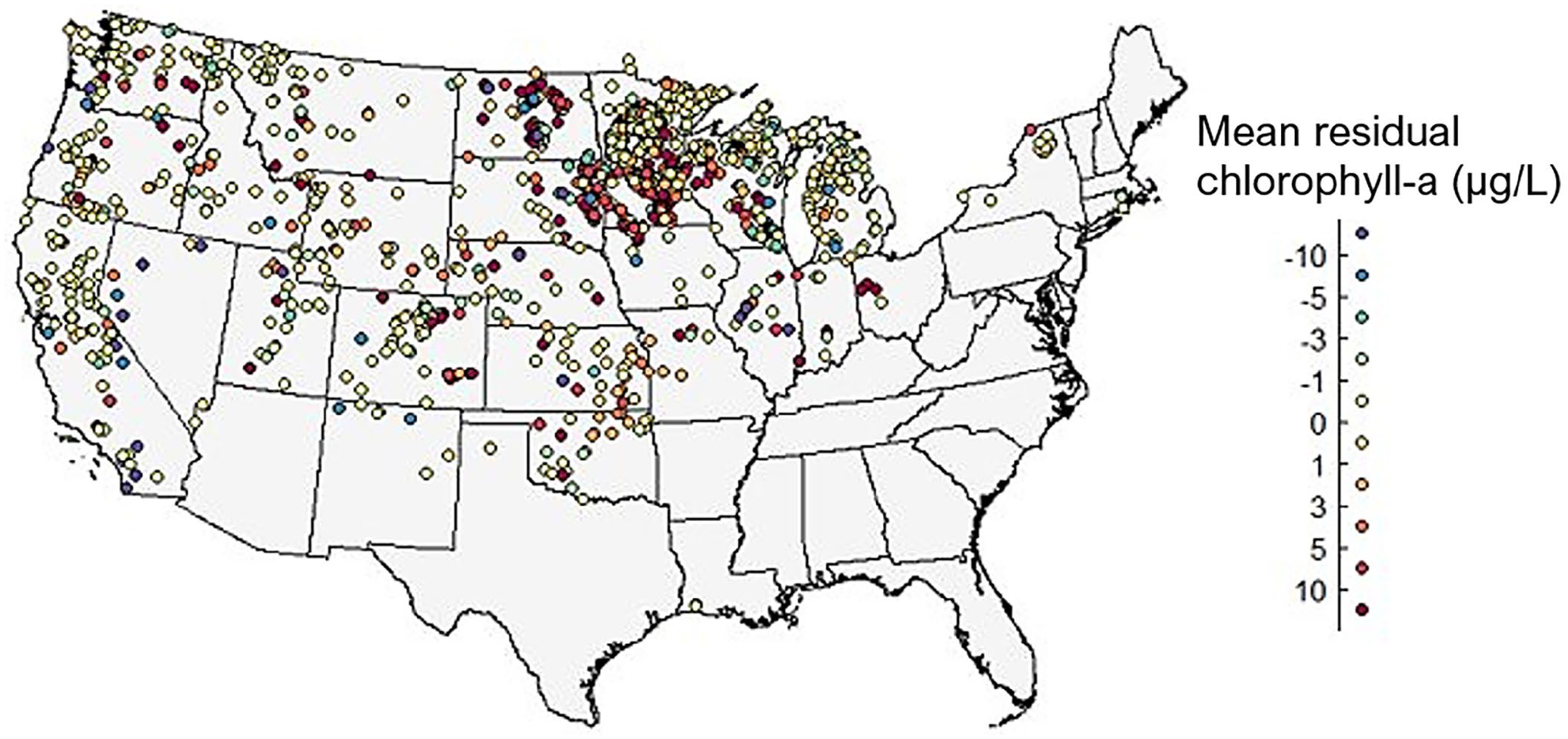
Maps illustrating changes in average residual surface chlorophyll-a concentrations for individual lakes impacted by smoke events. The number of lakes in each category is as follows: −10 μg/L, 31 lakes; −5 μg/L, 30 lakes; −3 μg/L, 37 lakes; −1 μg/L, 74 lakes; 0 μg/L, 339 lakes; 1 μg/L, 437 lakes; 3 μg/L, 84 lakes; 5 μg/L, 61 lakes; 10 μg/L, 88 lakes; > 10 μg/L, 85 lakes.

Spatially, lakes from across the CONUS exhibited increased average residual surface chlorophyll-a responses following intersection with smoke (Figure 5). The highest number of lakes (*n* = 755, 60%) experienced an increase in average surface chlorophyll-a and were observed across the CONUS. Eighty-five lakes (6.7%) had drastic increases in average residual surface chlorophyll-a of greater than 10 μg/L; many of these were clustered in the Upper Midwest. As noted above, this area experienced a high number of smoke days (Figure 1), impacted by both fires in the western U.S. and in Canada. Notably, only 40% (*n* = 511) of lakes analyzed experienced a decrease in average surface chlorophyll-a following intersection with wildfire smoke.

**FIGURE 6 |:**
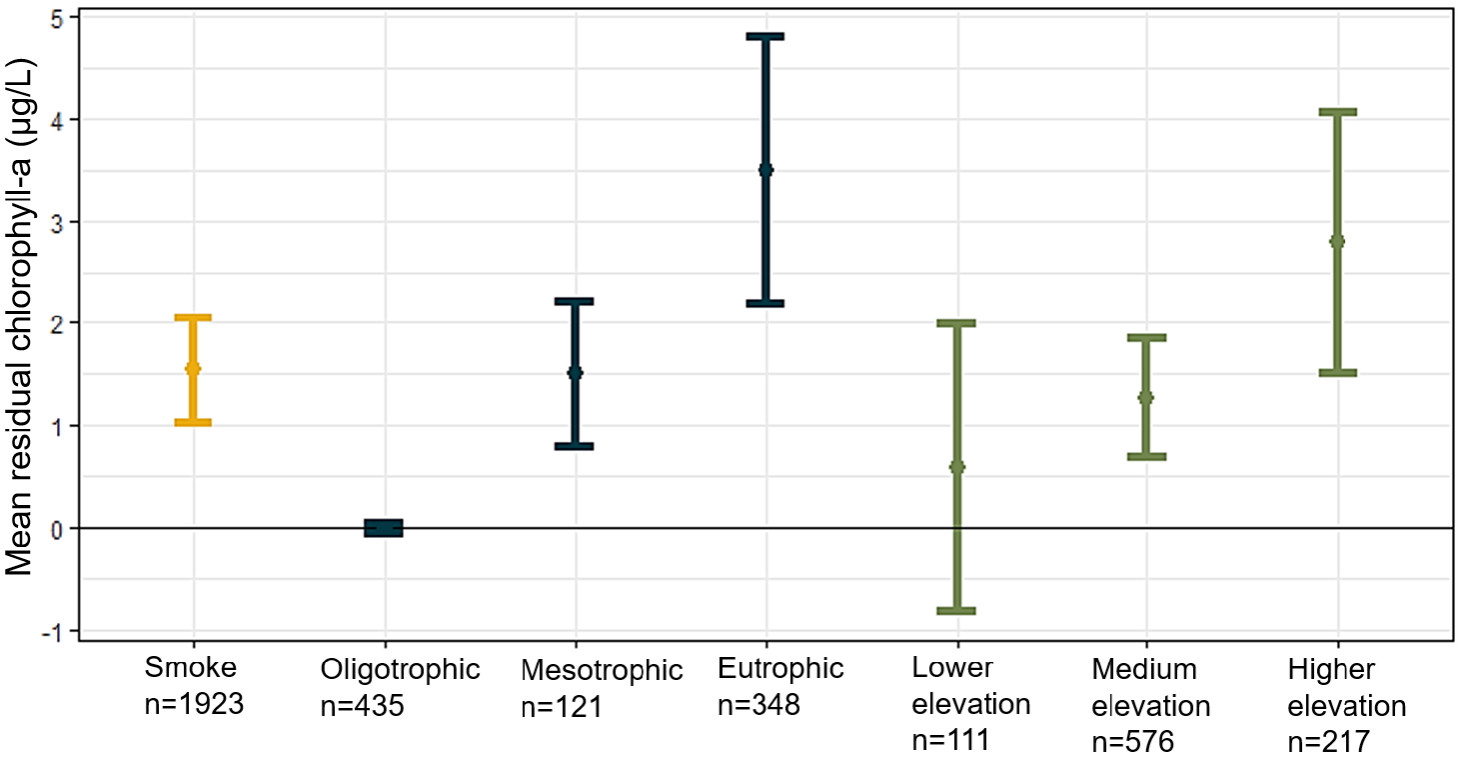
Mean residual surface chlorophyll-a values for smoke impacted lakes, lakes of different trophic status, and lakes of varying elevation. Trophic statuses were determined by EPA guidelines and are classified as follows: Oligotrophic (chlorophyll-a < 2 μg/L), mesotrophic (chlorophyll-a 2–7 μg/L), and eutrophic (chlorophyll-a > 7 μg/L). Elevation categories were determined by calculating the lowest, middle two, and highest quartiles of elevation from the population of lakes and are classified as follows: Lower elevation (< 255 m), medium elevation (255–538 m), and higher elevation (> 538 m). Bars represent the 95% confidence intervals around the mean response indicated by a circle. Data represented in this figure are shown in Table S4.

Lastly, we compared the weekly change in residual surface chlorophyll-a for lakes of different trophic status and elevation (Figure 6). Smoke impacted lakes had a higher and statistically significant increase in residual surface chlorophyll-a (1.56 μg/L, *p* < 0.05) compared to lakes not impacted by smoke (−0.3 μg/L), consistent with our findings in Figure 3. Eutrophic lakes experienced higher residual surface chlorophyll-a (3.50 μg/L; Table S4) compared to mesotrophic (1.50 μg/L) and oligotrophic lakes (0.00 μg/L). Likewise, lakes at higher elevation (> 538 m) also experienced greater increases in residual surface chlorophyll-a (2.79 μg/L) compared to lakes at medium (1.26 μg/L, 255–538 m elevation) or lower elevation (0.58 μg/L, < 255 m elevation).

## 4 | Discussion

Overall, surface chlorophyll-a significantly increased with both the presence of smoke and increasing smoke density in downwind lakes and reservoirs. This effect was relatively rapid and ephemeral, with surface chlorophyll-a increasing in many lakes in the 2 weeks following contact with smoke when compared to lakes without smoke, generally followed by a return to baseline conditions. These findings are similar to previous observations of aquatic effects related to high nutrient-containing wildfire smoke (Olson et al. 2023), though a much broader spatial scale is examined here. Changes in residual surface chlorophyll-a were mapped and identified across the CONUS. Geographically, there were relatively high responding lakes in most regions of the country, but many were concentrated in the Upper Midwest. Not only are there numerous lakes in this region, but this was also an area that experienced high numbers of smoke days in 2018 (Figure 1) due to western U.S. and Canadian wildfires. Finally, we observed greater changes in surface chlorophyll-a for lakes at higher elevation (> 538 m) and lakes with higher antecedent chlorophyll-a (i.e., eutrophic lakes).

Most fire and water quality studies to date have focused on post-fire movement of nutrients via waterways. A recent review found that nutrients typically increase in nearby waterways and stay elevated for several years following fire (Paul et al. 2022). In most cases, nutrient concentrations returned to starting levels within 2–4 years; however, nutrient levels in some fire-impacted streams have remained elevated up to 10 years post-fire (Emelko et al. 2016; Hauer and Spencer 1998; Rhoades et al. 2019). Several studies have reported increased primary productivity for up to 3 years following wildfire-influenced nutrient fluxes (Goldman and Jassby 1990; Kelly et al. 2006; Planas et al. 2000; Scrimgeour et al. 2001). These studies all focused on runoff as the mechanism by which nutrients from fire can enter local waterways and affect aquatic chemistry.

The effect of nutrient transport and deposition from wildfire smoke and its effects on algal blooms are an emerging area of research. Deposition of iron and particulates from wildfire smoke has been shown to enhance and alter algal blooms in marine ecosystems (Peng et al. 2024; Tang et al. 2021). Similarly, atmospheric nutrient deposition is increasing nutrient loading in lakes and streams (Stoddard et al. 2016), particularly in high elevation lakes (Camarero and Catalan 2012; Scholz and Brahney 2022). These studies provide growing evidence that wildfires likely account for another source of nutrients and may contribute to the reported nutrient shifts. Other studies have shown an increase in nutrient concentrations downwind from smoke events. For example, Fernandez et al. (2024) recently reported a sharp increase in stream phosphorus concentrations in the Finger Lakes region of New York coinciding with smoke from the Canadian fires in the summer of 2023. Similarly, a study in Montana reported a 5-to 60-fold increase in phosphorus and nitrogen concentrations in several streams downwind of fires in the first few days of the event (Spencer and Hauer 1991). They attributed the rise in nutrients to direct deposition of ash and dissolving of material into the waterbodies. Interestingly, nutrient levels returned to normal within several weeks of the fire. This rapid increase and decline in a short period of time mirrors the ephemeral bloom response reported in this study (Figure 4).

Relatedly, recent studies have noted smoke-influenced radiative effects that impact bloom activity. Wildfire smoke coverage has been shown to reduce incident radiation and heat transfer to mountain lakes in California (Scordo et al. 2022; Williamson et al. 2016), leading to an increase in primary production in shallow waters (Scordo et al. 2021). This same study found a decrease in chlorophyll-a across the water column for deep oligotrophic lakes, indicating a decrease in deep (benthic) productivity with wildfire smoke (Scordo et al. 2021). Smits et al. (2024) and Williamson et al. (2016) attribute both mechanisms (light effects and nutrient deposition) to changes in lake metabolic responses. While we cannot discern radiative effects from nutrient deposition in this work, we provide further evidence that wildfire smoke affects algal blooms in U.S. lakes and reservoirs.

Wildfire smoke can be mobilized and transported long distances in the air and across watershed boundaries, creating a more widespread zone of impact far beyond the burned area itself and extending outside of traditional fire-prone regions. The increasing frequency and magnitude of wildfires have profound implications for water quality managers, who must understand whether a lake intersects with smoke and then anticipate potential water quality responses.

Cyanobacteria blooms can occur naturally, particularly due to increased temperature (Paerl and Huisman 2008) and nutrient loading (Anderson et al. 2002). Since wildfire and elevated temperatures tend to be correlated, disentangling smoke effects from high temperature effects is difficult. We propose that wildfire-driven nutrient mobilization and smoke effects may be an additional stressor contributing to the formation of HABs given the proper antecedent conditions (e.g., elevated water and air temperatures) that are likely to coincide with wildfire activity.

In this study, surface chlorophyll-a concentrations generally increased with greater smoke density. Across all lakes, there were statistically significant changes in residual chlorophyll-a for lakes experiencing medium-and high-density smoke compared to lakes affected by low or no smoke. Moreover, fires—and their effects—are stochastic events, so variation in responses must be considered. Over 100 lakes exhibited a 1–5 μg/L increase in surface chlorophyll-a, and over 150 showed an increase above 5 μg/L coinciding with smoke events (Figure 5). Many of the highest responding lakes were located in the Upper Midwest, in areas with a high number of smoke days in 2018. Recently, other studies have noted an increase in medium-and high-density smoke days from 2019 to 2021 (Farruggia et al. 2024). However, to the best of our knowledge, water quality effects with changes in overhead smoke density have not been reported in the literature until this study.

In our analysis, lakes at higher elevations (> 538 m) were more susceptible to smoke-influenced changes in chlorophyll-a. While we did not have enough data to do a detailed study on mountain lakes (elevation > 1500 m), our results suggest that elevation, even below this cutoff, has a significant impact on chlorophyll-a response to smoke. A study of 29 mountain lakes in Oregon, U.S. found that nitrogen-fixing cyanobacteria were positively associated with lake phosphorus concentration, likely from a combination of geologic sources and atmospheric deposition (Jansen et al. 2024). Mountain lakes are highly valued for recreation and habitat for sensitive species due to often pristine conditions. Mountain lakes are also vulnerable to warming temperatures (Moser et al. 2019) and atmospheric deposition of nutrients (Brahney et al. 2015; Tipping et al. 2014). Therefore, the potential for HABs in high elevation mountain lakes is likely to rise in the future, making it crucial to understand the drivers of cyanobacteria blooms in these sensitive ecosystems.

Algal blooms impact drinking water and lake trophic status. Recommendations for drinking water sources are alert level 1 when chlorophyll-a concentrations are above 1 μg/L and biovolume (algal density) is above 0.3 μg/L, with additional confirmation that cyanobacteria are dominant in the phytoplankton community (Chorus and Welker 2021; Humpage and Cunlie 2021). Alert level 2 is recommended when chlorophyll-a concentrations are above 12 μg/L and biovolume is above 4 μg/L with cyanobacteria dominant. Concentrations at or above these thresholds indicate potential presence of cyanobacterial toxins and usually necessitate additional testing of drinking water supplies. Of the 2196 satellite resolvable lakes in the CONUS, 877 lakes (40%) have a drinking water intake location within 100 m (Coffer, Schaeffer, Foreman, et al. 2021). For aquatic health and welfare effects, EPA classifies lake trophic status according to chlorophyll-a concentrations present. Lakes with less than 7 μg/L are considered oligotrophic or mesotrophic, lakes with 7–30 μg/L are eutrophic, and lakes with greater than 30 μg/L are hypereutrophic (U.S. EPA 2009; Zhao et al. 2023).

Given these guidelines, increases in chlorophyll-a from wildfire smoke can be ecologically meaningful, particularly for medium- or high-density smoke events that represent average spikes of 1.6 and 1.2 μg/L, respectively. Increases in chlorophyll-a of this magnitude would trigger drinking water alerts and potentially change lake trophic status. In fact, we observed over 150 lakes with surface chlorophyll-a increases of 5 μg/L or more coinciding with smoke events. This suggests that smoke events can easily lead to cases where drinking water alerts are triggered and trophic status changes, especially if chlorophyll-a levels are already moderately elevated prior to the fire. Even a small increase in chlorophyll-a may push a lake into another trophic status (e.g., from oligotrophic to mesotrophic) if the lake is already close to the threshold. Whether a lake is under or over a given threshold could be important for water quality managers tracking trophic states of waters across the U.S. or a given region.

Besides chlorophyll-a standards, smoke could lead to exceedances of cyanotoxin guidelines, for which chlorophyll-a can serve as a risk metric. Cyanobacteria blooms can produce cyanotoxins such as microcystins (Dionysiou 2010) and cylindrospermopsin (Falconer and Humpage 2006), both of which have health advisory levels for safe consumption in drinking water in the U.S. (1.6 mg/L for microcystin (U.S. EPA 2015a) and 3 mg/L for cylindrospermopsin (U.S. EPA 2015b)). These toxins require costly purification, often by oxidation (Schneider and Bláha 2020) or ozonolysis (Hitzfeld et al. 2000), when present in drinking water supplies. For example, toxic algae blooms in Oregon’s Detroit Lake produced cylindrospermopsin and microcystin, which impacted drinking water for 200,000 people in the surrounding communities in 2018 (Dreher et al. 2022; Walton 2022). Cyanobacteria blooms can also affect secondary drinking water standards, such as taste and odor (Suffet et al. 2004). Other work has identified transfer of biological material and algal toxins from freshwater HABs to the atmosphere in PM_2.5_ (May et al. 2018; Olson et al. 2020; Shi et al. 2023), a key size range that affects human health via inhalation (Poschl 2005). These studies, combined with evidence of marine HAB toxin aerosolization (Van Acker et al. 2021; Vejerano et al. 2024), demonstrate another potentially important impact of freshwater HABs on public health.

In addition to human health impacts, algal blooms are an increasing threat to inland water quality and aquatic ecosystems (Brooks et al. 2016). Blooms can also lead to decreased dissolved oxygen levels (Chislock et al. 2013; Griffith and Gobler 2020; Skei et al. 2000), altered light and heat transport in water bodies (Skei et al. 2000), and negative impacts on biota (Paerl et al. 2001; Skei et al. 2000). Several studies have noted reductions in abundance or diversity of macroinvertebrates (Earl and Blinn 2003; Martens et al. 2019; Minshall et al. 2001; Minshall et al. 1995; Silins et al. 2014) and fish (Dunham et al. 2003; Rinne 1996) following fire-related eutrophication and cyanobacterial blooms. In turn, these can lead to negative impacts on fishing industries, recreation, and tourism (Ritzman et al. 2018).

## 5 | Limitations and Next Steps

As in all studies, there are limitations that should be considered when interpreting results. First, the use of remote-sensing data for both smoke plume detection and surface chlorophyll-a quantification contains inherent limitations. The smoke plumes from HMS are representative of smoke throughout the entire atmospheric column, not just at the surface. This may reduce the distinction between smoke and no smoke events if smoke plumes were high in the atmospheric column and did not interact with the lakes at the water surface (Liu et al. 2024), though recent studies suggest radiative effects from elevated smoke may still be applicable (Farruggia et al. 2024; Scordo et al. 2021, 2022; Smits et al. 2024; Williamson et al. 2016). Additionally, HMS satellite products could miss smoke due to cloud coverage, resulting in the mislabeling of smoke and no smoke days. Similarly, the satellite-based OLCI could have interference with smoke, glint, clouds, and cloud shadows. This could reduce the magnitude of surface chlorophyll-a measurements if smoke was consistently over a given lake during image acquisition. In addition, the OLCI only measures surface chlorophyll-a and is not indicative of chlorophyll in the entire aquatic column. The lakes analyzed herein were at least 300 m × 300 m (i.e., to be resolvable by OLCI), so results may be different (likely greater) for smaller aquatic systems. For example, (Smits et al. 2024) observed the greatest change in primary productivity for small, high elevation lakes in California during smoke exposure. Mixed land-water pixels were excluded from this analysis, though this can be where blooms accumulate due to wind advection. Accounting for these limitations would likely increase the magnitude of chlorophyll-a abundances in these waterbodies and the strength of the smoke effect reported here.

Other limitations include the lack of depositional flux estimates, lack of data for high elevation (> 1500 m) lakes, and trends across multiple fire years. Deposition flux estimates for nutrients were not attainable in the current work but represent an important next step in determining relationships between fire emissions and aquatic ecosystem effects. Future modeling work is needed to determine wildfire-influenced nutrient fluxes to water surfaces. Next, chlorophyll-a data were scarcely available for lakes > 1500 m, making inferences about high elevation, alpine lakes difficult. Finally, we focused exclusively on smoke and cyanobacteria data from 2018 because this year had increased Sentinel-3 measurements and frequent wildfire activity. This resulted in good spatial coverage over the CONUS, but we did not attempt to analyze temporal trends across multiple years. Follow-up work is needed to assess smoke impacts across multiple years, including potential accumulation of effects across years. As both wildfire (Abatzoglou and Williams 2016; Holden et al. 2018; Radeloff et al. 2018) and algal bloom activity (Mantzouki et al. 2018; Paerl and Huisman 2008; Smucker et al. 2021; Taranu et al. 2015) are anticipated to increase in coming years, further elucidating this relationship both temporally and spatially will become even more needed.

## 6 | Conclusions

In this study, we observed a significant correlation between the presence of wildfire smoke and increases in surface chlorophyll-a concentrations in U.S. lakes and reservoirs. Smoke events led to higher surface chlorophyll-a concentrations than expected based on seasonal patterns, while low or no smoke chlorophyll-a residuals were closer to zero. Surface chlorophyll-a responses increased significantly with higher smoke density, and concentrations rose across the CONUS after smoke events, with concentrations in over 150 lakes increasing by more than 5 μg/L in a period coinciding with smoke exposure. This suggests smoke can readily cause water concentrations of chlorophyll-a to exceed drinking water alerts (1 μg/L chlorophyll-a with presence of algal biomass). This evidence from over 2000 lakes, combined with our previous case-study performed in California (Olson et al. 2023), strongly suggests nutrient mobilization and radiative effects from wildfire smoke plumes impact aquatic ecosystems nearby and downwind from fire epicenters. Future studies could focus on examining this phenomenon across multiple fire years, performing targeted water sampling during and immediately after smoke intersections with lakes, and improved depositional modeling of fire-mobilized nutrients. Fire activity is projected to increase in the United States and many places globally. Our findings suggest the water quality implications of smoke-associated algal blooms are farther reaching than previously thought, extending beyond the burned watershed itself to impact national and regional scale water resources. We anticipate this research will help water quality managers anticipate, track, and respond to these potential effects to our waters.

## Supplementary Material

Supplement1

Additional supporting information can be found online in the Supporting Information section. Data S1: gcb470004-sup-0001-Supinfo.docx.

## Data Availability

A data repository containing all data and code for this analysis is available at https://sciencehub.epa.gov/sciencehub/datasets/4958, DOI: https://doi.org/10.23719/1532164.
