# Supplementary material for: Algal Blooms in Lakes Increase After Wildfire Smoke Events in the Contiguous United States
Source: Glob Chang Biol Commun. Author manuscript; Available in PMC 2026 May 5. (PMC13137383; doi:10.1002/gcb4.70004)
Supplement: Supplement1 [file NIHMS2157700-supplement-Supplement1.docx]

Supplemental material for: Algal blooms in lakes increase after wildfire smoke events in the contiguous United States

Nicole E. Olson^1^, Meredith M. Brehob^2^, Robert D. Sabo^1^, Irena Pavlovic^3^, Kathleen I. Shank^4^, Sam Penry^4^, Amalia M. Handler^1^, Michael J. Pennino^1^, R. Byron Rice^1^, Katie L. Boaggio^5^, Stephen D. LeDuc^1^

^1^U.S. Environmental Protection Agency, Office of Research and Development

^2^Oak Ridge Institute for Science and Education, hosted at U.S. Environmental Protection Agency, Office of Research and Development

^3^U.S. Environmental Protection Agency, Region 9

^4^Oak Ridge Associated Universities, hosted at U.S. Environmental Protection Agency, Office of Research and Development

^5^U.S. Environmental Protection Agency, Office of Air and Radiation


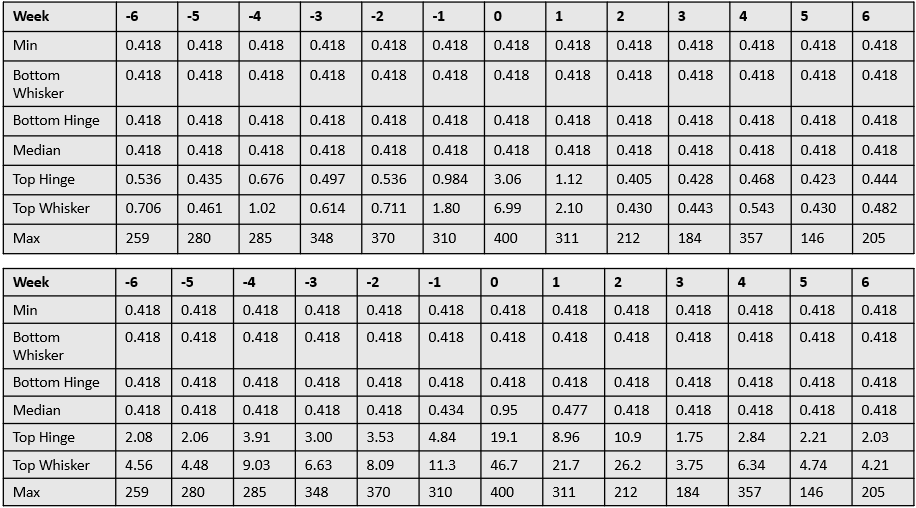


**Table S1.** Chlorophyll-a statistics shown in Figure 4. A negative number indicates weeks preceding a smoke event while week 0 represents the smoke event. The table on the top includes all data; the table on the bottom removed lakes with >80% of observations at the method detection limit to easier visualize trends.


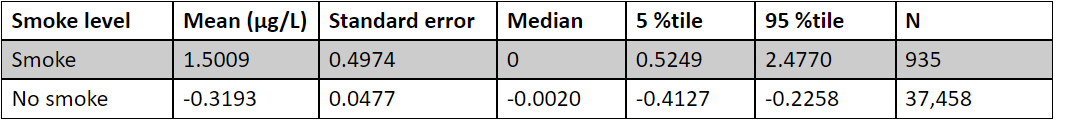


**Table S2.** Chlorophyll-a values resulting from spline regression of the smoke and no smoke event categories. Higher mean and median chlorophyll-a concentrations were calculated for smoke events compared to the no smoke controls. Smoke and no smoke categories are significantly different from each other.​

​


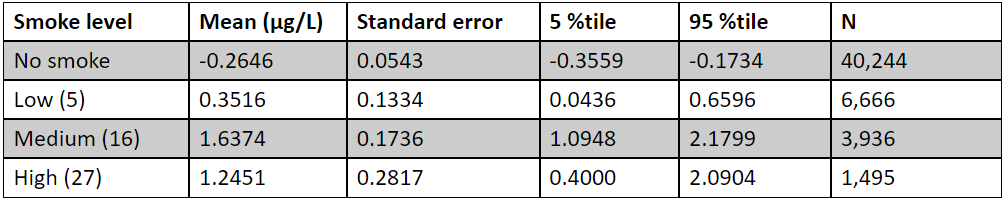


**Table S3.** Chlorophyll-a difference between predicted and observed values determined by the spline fit. No smoke and low-density smoke categories are significantly different from each other and from the medium- and high-density smoke categories. ​


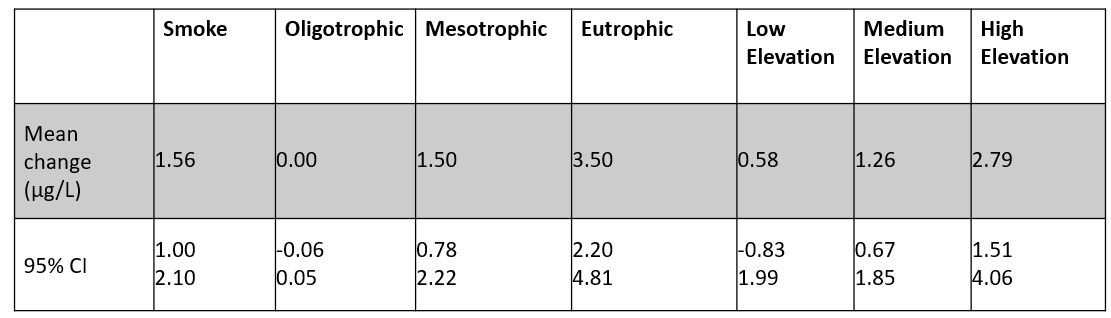


**Table S4.** Change in residual chlorophyll-a concentrations for smoke impacted lakes, and lakes with different trophic status and elevation shown in Figure 6. Tropic statuses were determined by EPA guidelines and are classified as follows: oligotrophic (n=435, chlorophyll-a <2 µg/L), mesotrophic (n=121, chlorophyll-a 2–7 µg/L), and eutrophic (n=348, chlorophyll-a >7 µg/L). Elevation categories were determined by calculating the lowest, middle two, and highest quartiles of elevation from the population of lakes and are classified as follows: lower elevation (n=111, < 255 m), medium elevation (n=576, 255–538 m) and higher elevation (n=217, > 538 m).


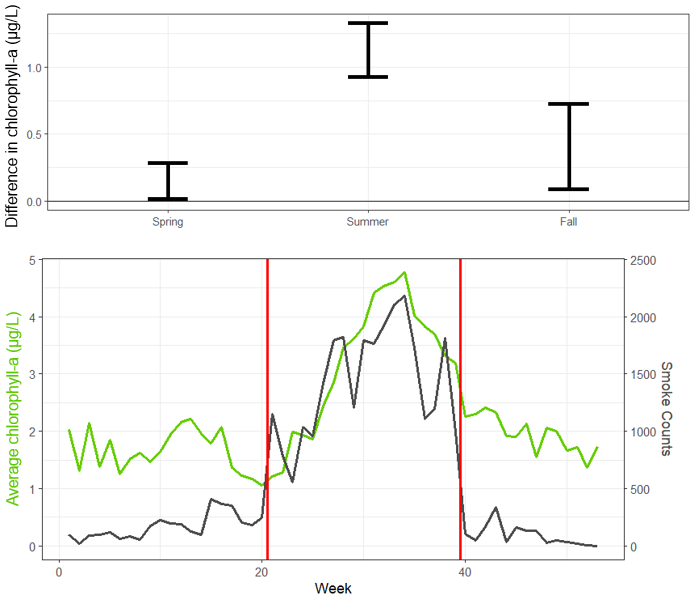


**Figure S1.** Average changes in chlorophyll-a concentrations and frequency of low- and medium-density smoke events for the year 2018. Red lines show rough seasonal breaks: spring (weeks 1-20), summer (weeks 21-39), and fall (weeks 40-53). The mean number of smoke days per lake was 19.9, with the median day of year for smoke coverage being 8-13-2018.


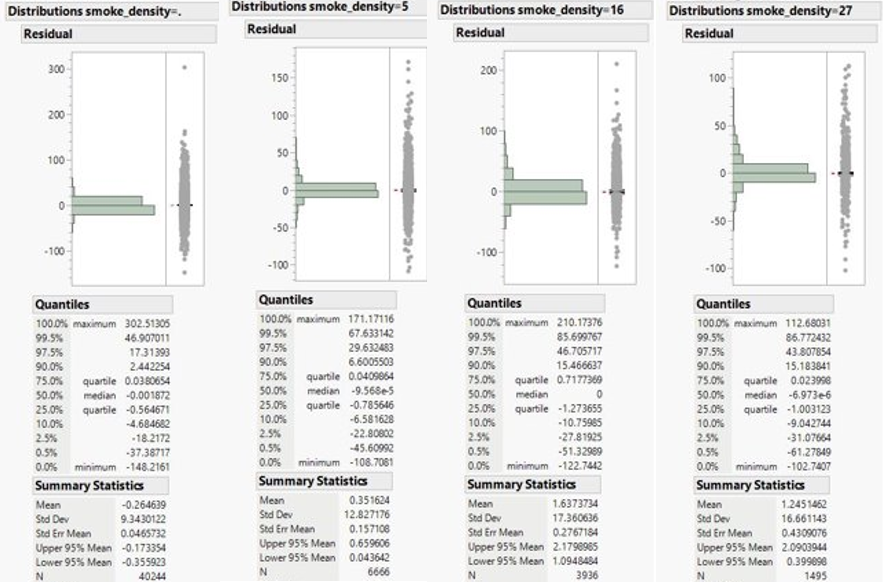


**Figure S2.** Histograms of chlorophyll-a residuals for no smoke and low-, medium-, and high-density smoke events (from left to right). Summary statistics are shown below each histogram. ​

​
